# Glymphatic System Dysfunction: A Novel Mediator of Sleep Disorders and Headaches

**DOI:** 10.3389/fneur.2022.885020

**Published:** 2022-05-19

**Authors:** Ting Yi, Ping Gao, Tianmin Zhu, Haiyan Yin, Shuoguo Jin

**Affiliations:** ^1^Rehabilitation and Health Preservation School, Chengdu University of TCM, Chengdu, China; ^2^School of Acupuncture and Tuina, Chengdu University of TCM, Chengdu, China; ^3^Hospital of Chengdu University of Traditional Chinese Medicine, Chengdu, China

**Keywords:** glymphatic system, headaches, sleep, neuropathology, aquaporin 4

## Abstract

Sleep contributes to the maintenance of overall health and well-being. There are a growing number of patients who have headache disorders that are significantly affected by poor sleep. This is a paradoxical relationship, whereby sleep deprivation or excess sleep leads to a worsening of headaches, yet sleep onset also alleviates ongoing headache pain. Currently, the mechanism of action remains controversial and poorly understood. The glymphatic system is a newly discovered perivascular network that encompasses the whole brain and is responsible for removing toxic proteins and waste metabolites from the brain as well as replenishing nutrition and energy. Recent studies have suggested that glymphatic dysfunction is a common underlying etiology of sleep disorders and headache pain. This study reviews the current literature on the relationship between the glymphatic system, sleep, and headaches, discusses their roles, and proposes acupuncture as a non-invasive way to focus on the glymphatic function to improve sleep quality and alleviate headache pain.

## Introduction

Sleep is vital to our body. Headache is a common pain complaint following poor sleep (1). Up to 70% of patients with chronic headaches also experience sleep disruption (2). Despite the common co-occurrence of these conditions, the relationship between headache and sleep is complex and poorly understood. Patients consistently report that poor sleep the previous night causes headache the next day (3), which indicates that poor sleep is a trigger for headaches and a higher frequency of headaches. However, sleep disturbances can be influenced by numerous factors. Although headache disorders have been suggested as a possible predisposing and perpetuating factor of sleep disturbances (4), the causal relationship regarding which occurs first, headache or poor sleep, remains a conundrum. To date, there have been some studies investigating the relationship, which suggests that the complex relationship between sleep and headache is bidirectional (4). However, a growing number of studies have suggested that sleep and headache share a common underlying etiology (5).

The glymphatic system (GS) is a newly discovered central nervous system (CNS) waste cleaning system that may offer a possible explanation for the relationship between sleep and headache (6). Its function is similar to the peripheral lymphatic system and relies on astrocytes; thus, the system is termed a “glia lymphatic” or “glymphatic” system, which represents a waste clearance and fluid pathway in and out of the brain (7, 8). When an individual experiences sleep disturbances, glymphatic exchange markedly decreases, and this blocks the interstitial fluid (ISF) outflow of excitatory substances or inflammatory chemicals from interstitial space, which affects metabolic balance (9, 10). Furthermore, a variety of headaches breaks the balance of the GS, leading to the accumulation of beta-amyloid (Aβ) and metalloproteinases (11), which increase the risk of sleep disruption. Therefore, GS dysfunction may be considered a common underlying pathophysiology of headache pain and sleep problems. We propose that regulating glymphatic function is critical for alleviating headaches and sleep disorders. In this review, we highlighted important discoveries made by human and animal research regarding the role the GS plays in sleep disorders and headaches. In addition, we presented a hypothetical model network to illustrate that GS dysfunction underlies the pathophysiology of sleep disorders and various types of headaches and provides implications for current and future research.

## Glymphatic System

**Figure 1:**
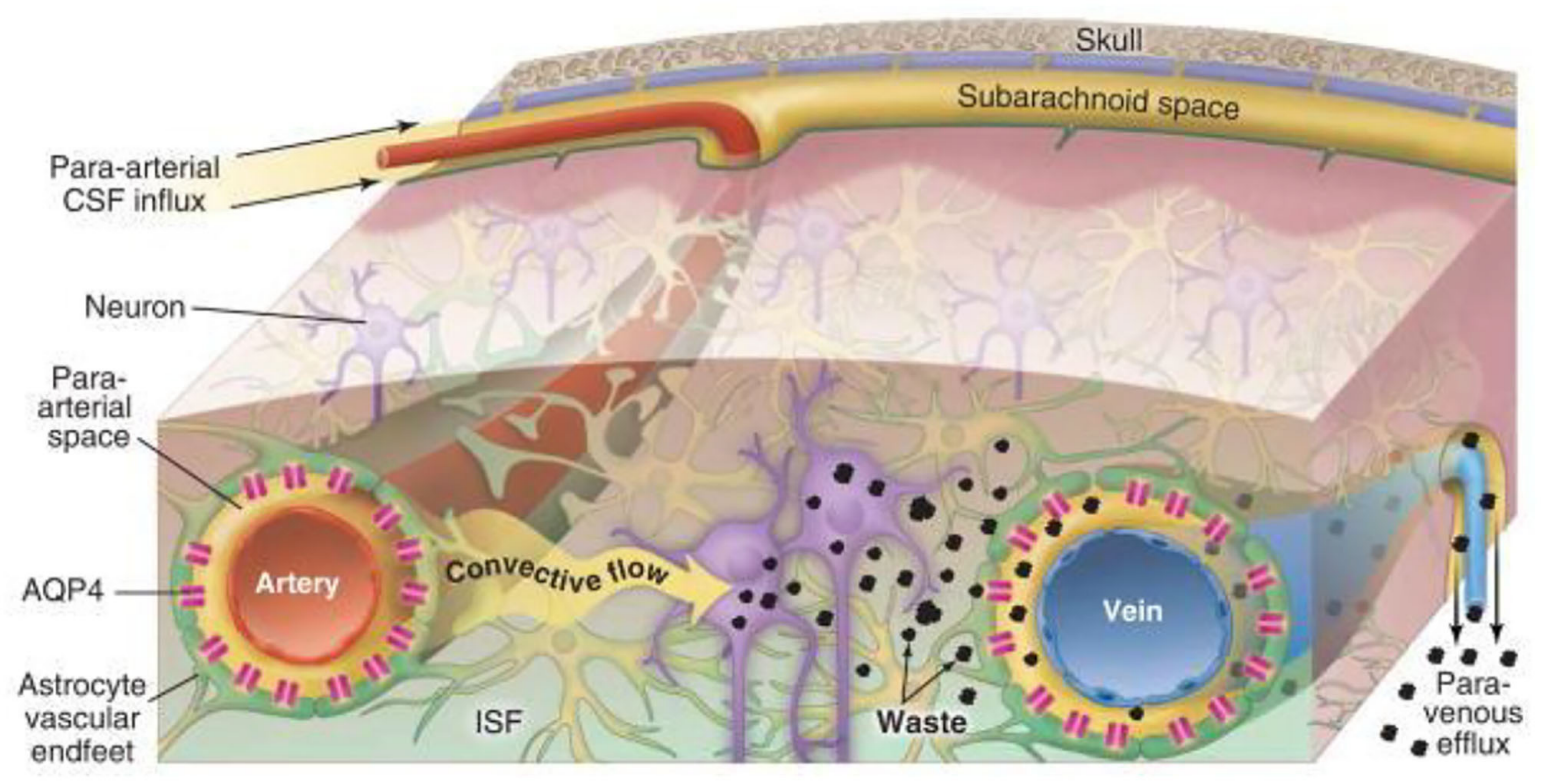
Schematic diagram of glymphatic transport in the brain (8). Cerebrospinal fluid (CSF) influx flows through the peri-arterial space *via* pulsation of the arterial wall and is then mediated by astrocyte aquaporin 4 (AQP4) in the brain parenchyma, which drives the interstitial fluid (ISF), whereby parenchymal metabolic waste substances exchange with the CSF to efflux into the peri-venous space. Eventually, the waste is cleared into the peripheral lymphatic system of the neck by arachnoid granulations and meningeal lymphatic vessels along with cranial and spinal nerve roots.

The GS is an effective waste clearance and fluid pathway for the CNS (for a detailed review see (8)). The current network model of GS transportation is described in Figure 1. The pathway consists of a periarterial influx route for cerebrospinal fluid (CSF) to center the brain parenchyma and a perivenous outflux route, which allows the clearance of ISF and extracellular solutes from the brain parenchyma (8). Extensive evidence has also shown that the bulk flow of CSF-ISF into the perivascular spaces (PVS) delivers glucose (12) and transports lipids, signaling molecules (13), and apolipoprotein E (14) for brain-wide energy metabolism. Moreover, the enhanced convective bulk flow of CSF-ISF for the removal of soluble proteins, waste, and excess extracellular fluid is dependent on astrocyte aquaporin 4 (AQP4) channels (15). Therefore, the two most critical elements during the process of continuous circulation and metabolism are the capacity of the PVS for holding metabolic fluid to transport and the mediating effect of AQP4 polarization to decrease resistance-enhancing bulk flow exchange on GS.

### The Main Structural Feature of the Glymphatic System

The para-arterial and para-venous spaces are collectively referred to as the PVS, which is the small tissue space that surrounds the cerebral arteries and veins. The inner wall is the vascular wall, and the outer wall comprises the basement membrane and the AQP4 expression in astrocyte endfeet, which wrap around and form the boundary of the space surrounding the blood vessel (16). The PVS is also known as the prelymphatic system and is considered an extension of the subarachnoid space, which is filled with CSF. The PVS narrows gradually at small arteries and arterial capillaries and eventually disappears. However, the outer wall of the blood vessel at the end remains surrounded by the basement membrane and astrocytes (17). The basal lamina provides low-resistance fluid space from which the CSF moves into the parenchyma through AQP4. The CSF convectively exchanges with the surrounding ISF in the brain parenchyma to expel and bind to metabolic waste and is subsequently cleared from the brain along with the surrounding space to the lymphatic system of the neck (15, 18). Thus, the key function of PVS is to enable the exchange of CSF and ISF. When PVS is constricted, it would increase resistance to convective fluid movement, suppress CSF influx, and accumulate metabolic wastes. Collectively, the loose fibrous matrix of the PVS provides an essential low-resistance pathway for the bulk flow, that is, a network of drainage channels that accelerates the flow of CSF-ISF and removes soluble proteins, toxic products, and metabolic waste within the GS from the brain.

Aquaporins are a family of water channels that are ubiquitously distributed among various tissues of the body. AQP4 is the most abundantly expressed aquaporin in the brain and participates in maintaining brain homeostasis (19). AQP4 is anchored to the dystrophin-associated protein complex *via* α-syntrophin, which is linked to laminin and agrin in the perivascular glial basement membrane by α-dystroglycan (20). Because of this complex molecular organization, AQP4 is unusually dense in the interface between the perivascular and interstitial spaces of the brain, which makes it easy to lower the resistance to aid CSF entry into the brain parenchyma and enhance CSF-ISF exchange, ultimately contributing to the exchange of solutes and metabolites in the bulk water flow (7). Research has shown that the polarization of AQP4 in healthy brains is highly distributed in the astrocyte endfeet of the outer wall of the PVS, which facilitates fluid influx into the parenchyma, fluid efflux out of the brain, and the elimination of brain metabolic waste (21, 22). When AQP4 channels are mislocalization or knockout, Iliff and colleagues found CSF influx significantly reduced relative to wild-type animals (7). As a result, we speculate that a significant reduction in the expression of AQP4 in astrocyte endfeet will lead to an impairment in the effective exchange of CSF-ISF, the accumulation of metabolic waste in the interstitium of brain tissues, and the gradual formation of plaque materials, which will eventually result in disease.

### Factors That Impact the Glymphatic System

The physiological function of the GS is affected by a variety of factors; however, the exact mechanism underlying its development remains unclear. In recent years, progress in the study of the GS with animal experiments and clinical trials has increased our understanding of the GS. It is currently believed that several factors affect the GS, including (1) arterial pulsatility, which is considered an important factor that drives the flow of CSF in PVS and subsequent CSF–ISF exchange. Ligation of the internal carotid artery causes CSF influx into the brain to slow, whereas enhanced arterial pulsatility following β agonist injection leads to an increase in CSF influx (23). (2) Sleep also affects the GS, in which the arousal level appears critical for governing glymphatic dynamics of CSF-ISF. There is an association between the sleep-wake cycle, water homeostasis, and effective clearance of pathological proteins. Hablitz et al. showed that glymphatic inflow was higher during slow-wave sleep (or ketamine/xylazine anesthesia) than during wakefulness and was positively correlated with delta brain waves and Aβ in the cortex (24, 25). The clearance rate of Aβ increased 2-fold, and the function of the GS increased significantly during sleep or under anesthesia (26). In order to further explore the role of sleep, the pioneering study of Fultz et al. suggested that global brain signals drive strong CSF movement associated with physiological modulations, especially during drowsiness or sleep in human (27), and global blood oxygen level-dependent (BOLD)–CSF coupling can be served as a marker for gauging glymphatic function (28). Intriguingly, this is the reason why recent studies have discovered that BOLD–CSF coupling is significantly weaker in those patients with AD (28) and PD (29) accompanying the accumulation of metabolic toxic wastes. (3) Finally, aging is a factor where brain aging has a significant impact on the function of the GS. A study compared the influx of the GS between aged and young mice and found that the influx of the CSF tracer in aged mice was 85% lower than that in young mice (10), which may be explained by meningeal lymphatic atrophy, arterial pulsatility impairment and perivascular AQP4 depolarization in the aging brain (30). In addition, other factors, such as inspiration (31) and body posture (32), have been shown to be related to the GS.

## The Glymphatic System In Various Types Of Headaches

### The Glymphatic System and Migraine

Migraine is a heterogeneous neurovascular disorder that affects people worldwide (33). Although the mechanism underlying chronic migraine is not fully understood, the trigeminovascular system has been shown to play a key role. Cortical spreading depression (CSD) and calcitonin gene-related peptide (CGRP) are recognized as key players in migraine pathophysiology (34).

**Figure 2:**
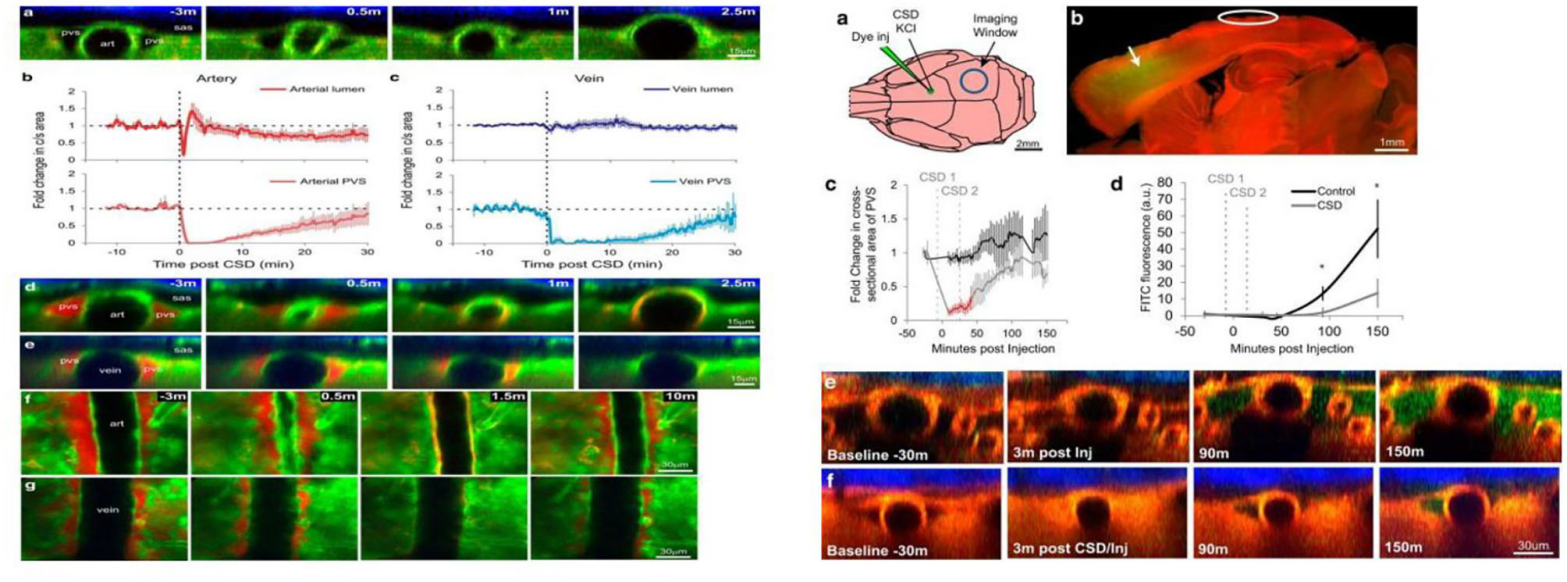
Cortical spreading depression (CSD) closes perivascular spaces (PVS), which impairs glymphatic flow, during migraine (11). The left panel illustrates the closure of the para-arterial space and para-venous space caused by the CSD over time *in vivo* two-photon. Specifically, the para-arterial and para-venous spaces close for 6 and 16 min, respectively, and partially reopen at 30 min, which causes the impairment of glymphatic flow that is represented in the right panel. The glymphatic flow over time (green dye) in CSD mice (right f) is lower than that in controls (right e).

Animal studies and the experience of spreading scintillations during migraine have shown that CSD, a pro-longed depolarization that spreads throughout the occipital lobe of the cortex, is the pathophysiologic mechanism underlyingly migraine aura. It is associated with transient disruption of ionic gradients, severe neuronal swelling, and neurotransmitter release, especially the local release of adenosine triphosphate, potassium, and hydrogen ions from neurons (33). A recent novel report demonstrated that CSD facilitates several minutes of the closure of PVS, a proposed exit route for ISF and solutes from the brain, which results in headaches by impairing glymphatic flow and preventing toxic substances to be cleared from the brain (Figure 2) (11). This study was the first to demonstrate that the GS is related to an abnormal cortical event that provokes a migraine attack. Moreover, based on the regulation of the width of the PVS by astrocytic endfeet, Rosic et al. further demonstrated that CSD is connected with the swelling of astrocytic endfeet using two-photon laser scanning microscopy in a different CSD mouse model (35). Taken together, these findings suggest that the closure of the PVS, which results in impaired clearance of excitatory and inflammatory waste along the paravascular pathways, plays a critical role in migraine pathogenesis.

The local inflammatory response is specifically triggered by CGRP, substance P, and pituitary adenylate cyclase-activating polypeptide from meningeal afferent fibers, following the vasodilation and microenvironment changes that occur during migraine (36). CGRP primarily mediates neurogenic inflammation and modulates nociceptive input (37). It is a primary neuropeptide involved in headaches, particularly migraine, and is an effective vasodilatory peptide that is widely applicable to migraine treatment (37). In 1990, Goadsby et al. first reported that CGRP was elevated in the extracerebral circulation during migraine (38) and that the CGRP level obtained from the jugular vein of migraine patients is higher than individuals without migraine (39). Newer drugs that target CGRP transmission along the migraine pain pathway have recently been approved by the United States Food and Drug Administration (40). CGRP is released by the trigeminal nerve innervating meninges, pia mater, intracerebral arteries, and the central projections in the spinal trigeminal nucleus at the level of the medulla (41). The regulatory mechanism of CGRP is well-understood; however, how it is metabolized after being released from the perivascular compartment into the blood remains unclear (41). It is expressed in C- and A-delta nociceptive nerve fibers and is widely released from perivascular trigeminal afferents, yet it cannot readily cross the blood-brain barrier (42). Thus, when it is released from nerve fibers, it initially cannot reach the vessels; however, the regulatory mechanism of CGRP needs to enter the perivascular space and subsequently into the CSF of the subarachnoid space. This is the reason why research has shown that the CGRP concentration in the CSF is five times higher than that in the plasma (43). Therefore, we propose that CGRP released from nerve fibers does not enter the vessels and instead diffuses into the surrounding perivascular space, which may be a continuation of the PVS of the penetrating arteries. This process may be mediated by the GS, which promotes the exchange and flow of CSF-ISF to maintain balance and eliminates CGRP (41). Therefore, the underlying pathological mechanisms of migraine pain may involve the GS, which offers insights into potential new antimigraine targets. Further, in a separate study, Hana and colleagues found that CGRP antagonists are not only a treatment for migraine but also for AD (44), which can reduce neuroinflammation and α-synuclein aggregation indicating a new therapeutic avenue for neurological disorders. While the study does not implicate that the mechanism is related to GS, it is a meaningful direction worth further exploring the exact role.

### The Glymphatic System and Post-traumatic Headache

Traumatic brain injury (TBI) is a devasting disorder that results in temporary or permanent neurological deficits and affects millions of people worldwide annually. In the past several decades, there has been an increase in the incidence of TBI globally, and this has coincided with a substantial increase in the incidence of post-traumatic headache (PTH). Globally, there are an estimated 69 million patients with TBI each year (45), whereas the 1-year prevalence for PTH is 21/100,00 (46). Moreover, PTH accounts for 4% of all headaches and is the second most common sequelae after TBI (47). The International Classification of Headache Disorders (ICHD) recently revised the diagnostic criteria for PTH in 2018 (48), although the criteria remain controversial. Nevertheless, PTH has attracted increased attention in recent years. The complex pathogenesis of TBI involves neuronal depolarization and the release of excitatory neurotransmitters, such as glutamate and aspartate, resulting in an increase in intracellular calcium, which induces dramatic changes in the metabolic state of the brain (47, 49). In addition, the accumulation of metabolic waste caused by abnormal metabolism in the brain is an important cause of PTH (50). Numerous recent studies have demonstrated the role of GS in TBI. In one study, Iliff et al. observed that the clearance of interstitial proteins and peptides from the brain parenchyma was impaired following TBI (51). Thus, it has been suggested that impairment in the clearance of CGRP and interstitial proteins and peptides may also underlie PTH. Furthermore, CGRP-mediated mechanisms could play a critical role in PTH pathogenesis. This was supported by an animal study that proposed blockade of CGRP as a potential therapy for managing PTH (52). Therefore, the role of the GS is maintaining balance, and preventing the CGRP level from becoming too high may be a promising treatment for PTH. In summary, we suggest that the GS is important for PTH, and CGRP is also a significant mediator of PTH. The clearance of waste from the brain due to glymphatic dysfunction is a likely cause of PTH (50, 53).

In addition, other researchers have found that TBI induces meningeal lymphatic drainage dysfunction, accompanied by morphological changes and increased intracranial pressure (ICP) (54). Intracranial hypertension (IIH) is a debilitating disorder characterized by ICP and causes chronic headaches that reduce the quality of life. Although the pathogenesis of IIH is poorly understood, recent evidence suggests that abnormal CSF absorption as a result of GS abnormalities is the mechanism underlying IIH (55). The venous outflow pathway plays an important role in maintaining the homeostatic balance of CSF as well as ICP. The glymphatic outflow pathway may serve as a compensatory pathway to regulate fluid homeostasis when the venous outflow pathway is dysfunctional. Thus, CSF absorption anomalies cause increased ICP, which contributes to the pathophysiology of IIH. Enhancing the function of the GS to accelerate CSF circulation may be a promising method to relieve headaches induced by IIH.

## The Glymphatic System and Sleep Disorders

Sleep is important to our bodies. Brain excitability, memory consolidation, and immune functions can become impaired following sleep disturbances (56). Rapid eye movement (REM) sleep and periods of non-REM (NREM) sleep are two metabolic and electrophysiological phases with distinct functions during sleep. The NREM sleep phase is subdivided into three stages: N1, N2, and N3. Because of the presence of slow EEG waves during N3, NREM is also called slow-wave sleep. Brain waves during REM sleep are similar to those during the awake state and are characterized by rapid eye movements. At the beginning of sleep, individuals enter the NREM sleep phase, whereby EEG exhibits slow waves, and subsequently, they enter the REM sleep phase; these phases alternate in a cyclic fashion all night (57–59).

Physiological changes in the organs and functions of the body during sleep occur as a result of changes in nervous system function. Experimental and epidemiological evidence has demonstrated a strengthening of the relationship between neurological decline and sleep problems; these conditions frequently co-occur (60). Sleep disorders have been linked to the development of neurogenerative diseases. Poor sleep impairs cognition, exacerbates depression and anxiety symptoms, and predisposes people to dementia, particularly Alzheimer's disease and Parkinson's disease (10, 61). Although the specific mechanisms underlying such effects are unknown, the involvement of the abnormal clearance of metabolic waste during sleep has been suggested (15). Our brain only weighs approximately 3 lbs, yet its high energy demand accounts for 40% of the body's total energy consumption. Moreover, cerebral oxygen consumption reduces by only 20% during sleep (5). Thus, such a high energy demand generates a higher amount of potentially toxic protein waste than that generated by other organs. However, the lack of lymphatic vasculature to remove large amounts of waste from the brain parenchyma is problematic. Further research on the GS that is focused on this issue will advance our understanding of the relationship between the GS and sleep disorders (62).

**Figure 3:**
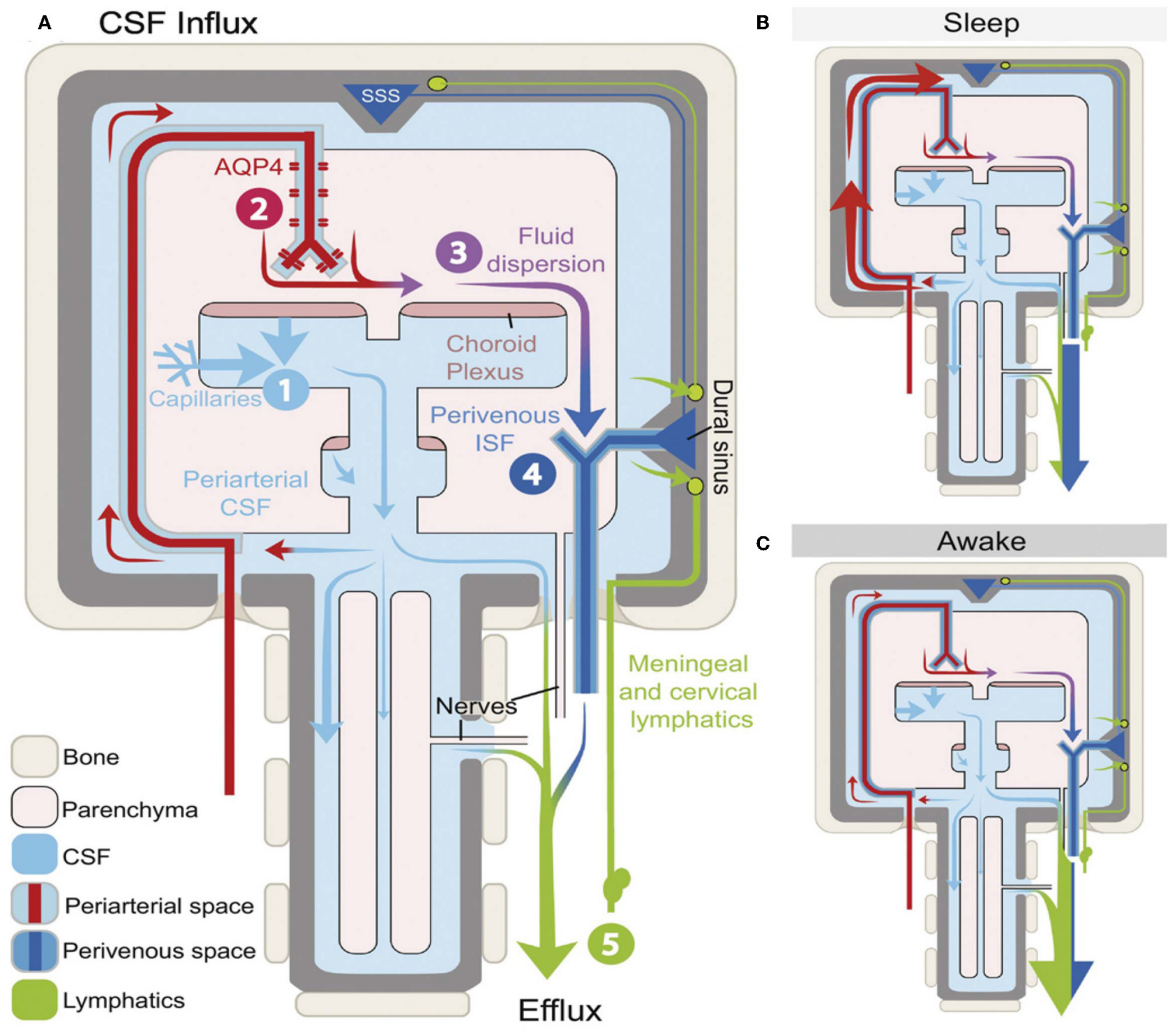
Schematic outline of glymphatic system (GS) function during different arousal states (64). The influx and efflux of the fluid transport pathway from GS **(A)** and its function varies during different arousal states. A glymphatic clearance occurs primarily during sleep via the enlargement of the periarterial space and the perivenous space to promote the glymphatic flow **(B)**. However, function decreasing in the GS results in the accumulation of metabolic waste, which builds up in the awake brain and mostly depends on lymphatic vessels to exclude **(C)**.

The most significant function of the GS is particularly active during sleep (63), whereby potentially toxic neural waste substances that accumulate during wakefulness are cleared *via* the GS (Figure 3) (24, 64). It is thought that the cell volume decreases during sleep, which expands the size of PVS, and this facilitates the influx of CSF into the peritubular space for material exchange and metabolic waste removal (10). Animal experiments using intravital 2-photon microscopy in mice showed that glymphatic clearance is decreased by 90% during wakefulness, while protein clearance in the intima of the brain doubles during sleep (24). In addition, Aβ levels in circadian-fluctuating CSF have been reported to significantly increase during wakefulness and decrease during sleep, especially during NREM sleep. To further understand the effects of sleep deprivation on Aβ clearance, a clinical trial in 20 healthy controls who underwent 31 h of sleep deprivation showed that Aβ was increased by 5% in the hippocampus, the parahippocampus, and the thalamus (65). Moreover, a recent study that tracked the REM phase in patients who experienced poor sleep found that the index of diffusion tensor imaging analysis along the perivascular space was significantly lower in patients than in healthy controls, which indicated glymphatic dysfunction (66). Thus, we speculate that the GS is intimately linked to various aspects of sleep.

### Sleep Position

Gravity can affect the flow and distribution of blood in the brain. Therefore, sleep position may be an important factor that affects the clearance of waste products from the brain. In a clinical study, Daniel et al. reported a significant association between sleeping in a supine position for more than 2 h/night and the development of neurodegenerative diseases. Interestingly, head position in a supine position during sleep can affect the clearance of neurotoxic proteins from the brain, which may trigger the development of neurodegenerative diseases (67). In another study in anesthetized rats, the transport and clearance efficiency of the GS was higher in the lateral position than in supine and prone positions, which corresponds to rats' preference for lateral decubitus behavior during rest and sleep under physiological conditions (32). Moreover, humans are also accustomed to lying in a lateral position during sleep. Thus, perhaps this is an effective way to prevent neurodegenerative diseases. Although there have not been any clinical trials investigating this idea, the potential mechanism warrants exploration.

### Sleep-Wake Cycle Alterations

The suprachiasmatic nucleus of the hypothalamus controls the sleep-wake circadian rhythm (68). Abnormal circadian rhythms and altered sleep-wake patterns often coexist. Patients who have trouble falling and staying asleep at night often experience excessive daytime sleepiness. Although sleep plays an important role in glymphatic function as discussed above, research has also shown that glymphatic function depends on not only the arousal state but also the daily rhythm. This explains why glymphatic influx and interstitial fluid clearance in mice are better at noon when they are asleep (69). To further explore whether its own circadian rhythm causes the function of the GS to change or the environment change causes the light/dark cycle. Lauren et al. observed circadian behavior from constant light (LL) in mice by continuous activity monitoring, and there is no significant change in the circadian system under 10 days in LL. Subsequently, these mice were exposed to LL for 10 days. Results showed that glymphatic influx, waste clearance, and fluid drainage persisted, which supports the hypothesis that the circadian oscillations that affect the GS are endogenous, not by light cycle (70, 71).

## Glymphatic System is a Possible Key in the Bidirectional Relationship Between Sleep and Headaches

Sleep disturbances and headaches occur throughout life. Their relationship is interdependent and often follows a vicious cycle. Headaches may occur during sleep, after sleep, or at different stages of sleep, whereas poor quality, short duration, inappropriate timing, and inappropriate sleep behaviors can also trigger headaches. Recently, the ICHD-3 described the relationship between headache and sleep disturbances, which included sleep-related headache disorders, such as migraine, cluster headache, chronic paroxysmal migraine, sleep-onset headache, and secondary headache (72). Some headache prevalence studies showed that tension-type headache (TTH) is larger than migraine worldwide, but migraine is closely linked to sleep disturbance (73). 48–74% of patients with migraine thought poor sleep is a predictor of headache, while patients with TTH only account for 26–72% (74). In patients with migraine, sleep disturbance manifested as insomnia, daytime sleepiness, obstructive sleep apnea, and parasomnia (night terrors, somnambulism) (75); Insomnia was most associated with migraine compared with other types of sleep disorders. The prevalence of insomnia in Korean patients with migraine is 25.9% (76). A prospective study has shown that patients with insomnia have higher risks of migraine after 11 years (77). The increased headache frequency in patients with migraine is associated with short sleep duration and poor sleep quality (78). Although the pathogenetic relationship between sleep and migraine is still not completely understood, they are commonly affected by potential factors such as high levels of stress, anxiety, and depression during the day, which are significant triggers of poor sleep and headache during the night. Interestingly, this is the reason why fatigue, weariness, and yawning typically precede headache attacks during the day. Thus, we concluded that headache promotes sleep disturbances, but sleep disturbances can also precede and trigger headaches.

**Figure 4:**
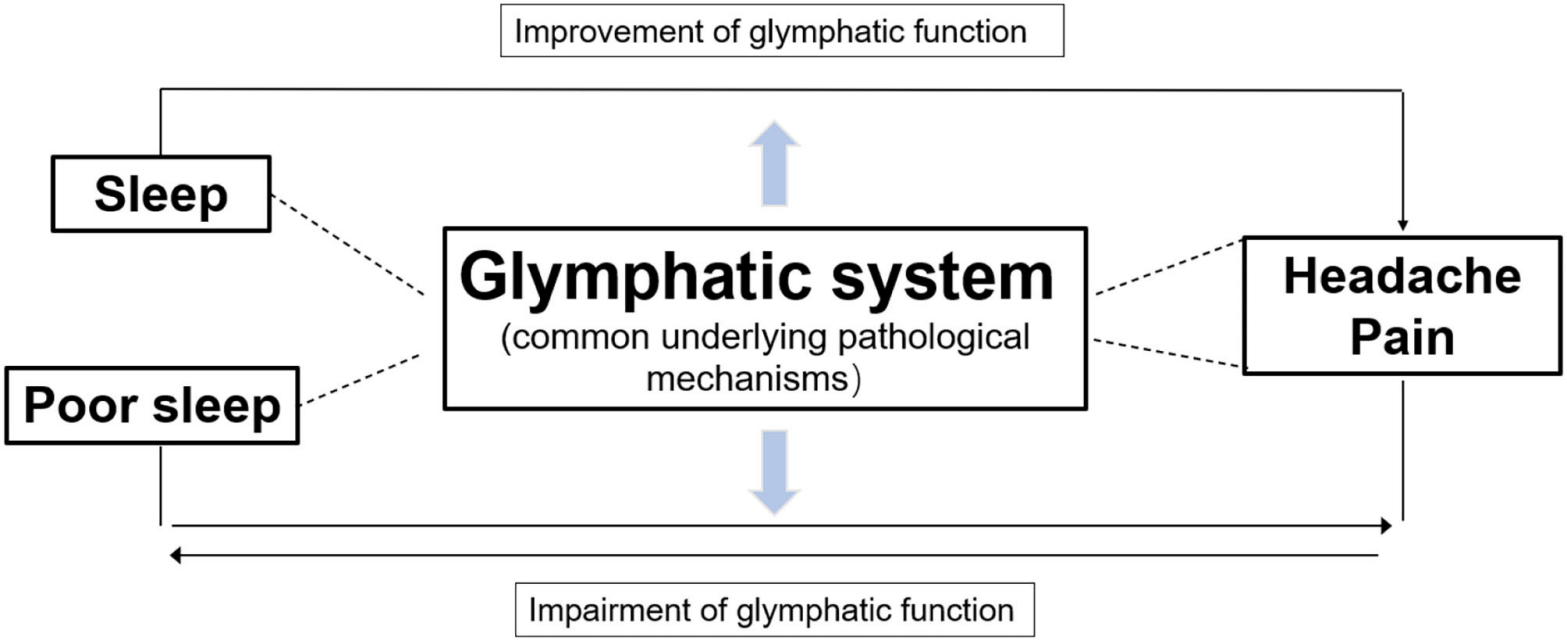
Diagram illustrating the relationship of three parts. GS dysfunction is a common underlying pathological mechanism of sleep disturbances and headache pain. Glymphatic flow, the clearance of waste products, and nutrient transportation can be improved during sleep, which may alleviate headaches. In contrast, poor sleep and headaches affect each other and may be connected indirectly, whereby both impairing glymphatic function, which further exacerbates both conditions.

Glymphatic dysfunction has been put forward as a common pathogenic mechanism of sleep disturbances and headaches (Figure 4). Sleep has been shown to play a major role in brain homeostasis and waste clearance *via* the GS (11). However, another study demonstrated that sleep deprivation reduces glycogen breakdown, which may ultimately contribute to CSD, rise the extracellular K^+^ levels activating inflammatory pathways, and impair glymphatic transport causing glucose or lactate transporter deficiency; subsequently, this forms a vicious cycle in the cortex that leads to migraine pathophysiology (79). Further, norepinephrine (NE), which regulates glymphatic function, is also an important factor that affects sleep and migraine. On the one hand, NE regulates the sleep-wake cycle of glymphatic exchange and the volume of extracellular perivascular space (80). Arousal causes a burst of NE release, which turns the GS off and increases the resistance of fluid transport. On the other hand, reports have shown that rats produce headache behaviors when NE is applied to the dura mater (81), which may explain why propranolol is one of the most effective first-line medications used for migraine prophylaxis. Therefore, GS may provide a tantalizing link between sleep disturbance and headache, and glymphatic dysfunction may be a common pathogenic factor.

There are direct and indirect factors that could explain how GS affects headache and sleep. They may directly influence each other *via* the GS; for example, sleep can decline NE levels from the locus coeruleus, thereby increasing the size of the interstitial space, lowering the resistance to convective fluid movement, and promoting CSF influx and interstitial solute efflux, thus it can improve the closure of PVS by CSD to alleviate the symptoms of migraine (11, 24); pain may directly block GS function by altering neuronal function, which in turn, causes sleep disturbances. Alternatively, there may be an indirect connection, whereby sleep disruption causes the accumulation of a large amount of metabolic waste, which triggers neuroinflammation and impairment of the GS, inducing intense headache symptoms (6, 50, 53). Thus, improving sleep quality can improve GS function by enlarging PVS, increasing CSF-ISF exchange, and promoting metabolic waste removal to relieve headaches.

Currently, effective and widely available treatments are limited for patients who experience headaches and sleep problems. Therefore, there is an urgent need to develop more effective and safe treatments. Perhaps treatments that increase CSF-ISF exchange and glymphatic clearance would improve sleep or reduce headache pain. Acupuncture is widely used clinically in patients with headaches and sleep disorders (82, 83). It is well-tolerated with little risk of serious adverse effects. Do non-pharmacological treatments, such as acupuncture, improve sleep and reduce pain? Moreover, do their effects correlate with the glymphatic function? Extensive evidence has shown that acupuncture plays an important role in reducing NE and inhibiting inflammation *via* multiple mechanisms (84, 85) and accelerating glymphatic clearance (86), which suggests that it may be an effective method for reducing the severity of headache pain while simultaneously improving sleep quality. Although this is a thought-provoking idea, validation in a larger prospective study is needed.

In conclusion, GS is a recently discovered waste clearance system for the CNS that helps remove metabolic waste products from the brain to maintain metabolic homeostasis. Impairment of the GS is observed in various neurological diseases, which include sleep and headache disorders. There is a close relationship between sleep disorders and headache disorders. Some headache disorders are significantly affected by lack of sleep; conversely, headache disorders can trigger sleep disturbances. Thus, we proposed a mechanistic model network of the glymphatic dysfunction to explain the bidirectional relationship between sleep and headache disorders and hypothesized that GS dysfunction is a common pathological mechanism of the two conditions. Although there is growing evidence to support the model, it is currently a theoretical framework, which still has some uncertain problems to be verified. For example, AQP4 is an important part of GS. When the perivascular AQP4 water channel is mislocated, it will contribute to the impairment of glymphatic flows. Recent research highlighted the role of AQP4 in human sleep-wake regulation (87). However, the trigger roles of AQP4 depolarization are still unknown when a patient is under headache attack after poor sleep. Further, sleep disturbance and headache are affected by many factors, for example, depression and anxiety are strong predictors in those with sleep and headache problems. Although depression is thought to decrease glymphatic function (88), is there any possibility that depression affects sleep and headaches by reducing GS function? Meanwhile, the brain structure (hypothalamic and brainstem) and neurotransmitters (melatonin and adenosine) have been hypothesized as an important pathological mechanism of sleep disturbance and headache, but there is a certain knowledge gap about whether these pathological mechanisms are related to GS. At last, the glymphatic system is a novel mechanism that has been recently discovered. It is still controversial and needs further exploration to pave the way for future research. In the future, we suggest identifying the key element that controls glymphatic exchange to maintain metabolic homeostasis, which would enable the development of preventive and therapeutic approaches for headaches and sleep disorders. As a non-pharmacological treatment, acupuncture may be promising as a non-invasive therapy. Furthermore, a better understanding of the pathological mechanisms underlying GS dysfunction may give rise to novel therapeutic strategies.

## Author Contributions

TY, SJ, and HY are responsible for the conception and design of the article, data collection and arrangement, and writing the paper. PG is responsible for the data collection. SJ, HY, and TZ are responsible for the revision, quality control, and review of the article. All authors contributed to the article and approved the submitted version.

## Funding

National Natural Science Foundation of China Youth Science Fund Project (82004342). Chengdu University of Traditional Chinese Medicine 2021 Xinglin Scholars Disciplinary Talents Scientific Research Promotion Project (QJRC2021006). Fund of Promotion Plan of The Affiliated Hospital of Chengdu University of Traditional Chinese Medicine (21-Q24).

## Conflict of Interest

The authors declare that the research was conducted in the absence of any commercial or financial relationships that could be construed as a potential conflict of interest.

## Publisher's Note

All claims expressed in this article are solely those of the authors and do not necessarily represent those of their affiliated organizations, or those of the publisher, the editors and the reviewers. Any product that may be evaluated in this article, or claim that may be made by its manufacturer, is not guaranteed or endorsed by the publisher.
